# Prevalence of p-16 Positive Laryngeal and Pharyngeal Tumors in Nepalese Population: A Hospital based Cross-sectional Study

**DOI:** 10.31729/jnma.5132

**Published:** 2020-09-30

**Authors:** Bigyan Raj Gyawali, Kunjan Acharya, Ravindra Sapkota, Dharma Kanta Baskota, Bimal Kumar Sinha

**Affiliations:** 1 Department of ENT-Head & Neck Surgery, Institute of Medicine, T.U. Teaching Hospital, Kathmandu, Nepal; 2 Sikhar Biotech Company, Lalitpur, Nepal; 3 Grande Int'l Hospital, Kathmandu, Nepal

**Keywords:** *immunohistochemistry*, *p16*, *tonsils*, *tumor*

## Abstract

**Introduction:**

P16 overexpression is considered as a good prognostic marker for oropharyngeal squamous cell carcinoma. However, there are very few literatures on the prevalence and outcomes of p16 overexpression in non-oropharyngeal squamous cell carcinoma and benign head and neck tumors. The aim of our study was to estimate the hospital based prevalence of p16 positive laryngeal and pharyngeal tumors and to compare it with the prevalence of p16 expression in the non tumor tissue (tonsils).

**Methods:**

This was a descriptive cross-sectional study. Cases of all genders >15 years presenting with malignant or benign tumors of larynx and all the subsites of pharynx were included in the study for evaluation of p16 expression by immunohistochemistry. Tonsillar tissue of cases undergoing tonsillectomy for recurrent acute tonsillitis were taken as non-tumorous tissue to evaluate for p16 expression.

**Results:**

A total of 48 cases were included in our study with 24 cases having different tumors of head and neck region and 24 cases having recurrent acute tonsillitis who were kept under non-tumor group. Eight cases (33.3%) in the tumor group showed positive stain for p16 in IHC. In non tumor group, 7 cases (29.1%) showed positive IHC staining for p16.

**Conclusions:**

P16 expression can be present in both benign and malignant tumors of various subsites of head and neck region and also in tonsillar tissue affected by inflammation.

## INTRODUCTION

Squamous cell carcinoma (SCC) is the commonest variant of carcinoma in head and neck region having an incidence of >5,00,000 cases each year globally.^1^ With tobacco and alcohol consumption being recognized as the most common risk factors for majority of head & neck SCC (HNSCC), Human papilloma virus (HPV) also has now emerged as an independent risk factor specially for oropharyngeal SCC (OPSCC).^2^ Apart from these factors, p16 protein is also related to the pathogenesis of HNSCC.^3^ P16, also called p16^INK4A^, is a protein that inhibits cyclin dependent kinase, there by slowing the progression of cell cycle from G1 to S phase.

P16 overexpression is considered as a good prognostic factor for OPSCC. While mechanism of p16 overexpression is clearly understood in HPV +ve tumors, its overexpression in HPV-ve tumors still remains unclear.^3^ Also, there are very few studies on the prevalence and outcomes of p16 overexpression in non OPSCC and benign head and neck tumors.

The aim of our study was to estimate the hospital based prevalence of p16 positive laryngeal and pharyngeal tumors and to compare it with the prevalence of p16 expression in non tumor tissue (tonsils).

## METHODS

This was a descriptive cross-sectional study conducted at Ganeshman Singh Memorial Academy for ENT-Head and Neck Studies, Maharajgunj Medical Campus, Tribhuvan University Teaching Hospital, Kathmandu, Nepal. Study was carried out from January 2019 to January 2020. Ethical approval from the Institutional Review Committee was taken. Cases of all genders >15 years presenting with malignant or benign tumors of larynx and all the subsites of pharynx were included in the study. Biopsy was taken under local or general anesthesia depending upon the accessibility of affected sites. Tissues for histopathological examination and for evaluation of p16 were separated. Cases refusing to give consent were excluded. Tonsillar tissue of cases undergoing tonsillectomy for recurrent acute tonsillitis were taken as non tumor tissue to evaluate for p16 expression. A whole sampling was done with all the cases meeting the inclusion criteria during the study duration being included in the study. Our total sample size was 51, of which three cases were excluded for not having adequate sample for IHC analysis. Of the 48 cases, 24 were in tumor group and 24 in non-tumor group. For all the cases, histopathological confirmation and immunohistochemistry (IHC) were done side by side. As per our research hypothesis, laryngeal and pharyngeal tumors will express p16 whereas, non-tumor tissue, i.e. tonsils, won't express p16. To avoid the observation bias, the technician doing the IHC analysis was blinded regarding the nature of the tissue sample.

For IHC, tissue fixation was done with ten percent formalin following which paraffin embedding was done. Sectioning with microtome to five micrometer thickness sections was done. The sections were transferred to glass slides following which deparaffinization and rehydration was done. Antigen retrieval was done by incubating in autoclave (15lbs) for three minutes. Endogenous peroxidase activity was blocked by incubating sections in three percent H2O2 solution in methanol at room temperature for ten minutes. Primary antibody i.e. Goat Anti-CDKN2A (isoform 3) antibody (EB07092) was applied to the sections on the slides following which horseradish peroxidase-conjugated secondary antibody i.e. Rabbit anti-Goat IgG antibody HRP Conjugated (EB2ND-001-HRP) was applied to the sections on the slides. 3,3'-Diaminobenzidine(DAB) substrate solution was applied to the sections. Counterstaining was done by applying 100 μl Hematoxylin working solution (ten times diluted) to the slides. Slides were then dehydrated with ethanol and xylene. Evaluation for staining was done under microscope. Detection of any cytological or nuclear staining was considered p16 positive.

## RESULTS

A total of 48 cases were included in our study with 24 cases having different tumors of head and neck region and 24 cases with recurrent acute tonsillitis who were kept under non tumor group. Of the total cases, 19 were male and five were female in tumor group. There were two cases of papilloma oropharynx, two cases of carcinoma (ca) oropharynx, two cases of ca hypopharynx, five cases of laryngeal papilloma, and 13 cases of ca larynx. Of the cases with recurrent acute tonsillitis, 13 were male and 11 were female. Majority of cases in tumor group were above 40 years, while all cases in non-tumor group were below 40 years (Table 1).

**Table 1:** Distribution of site of pathology and age in cases with positive p16 expression.

Site	Pathology	Total no. of cases (n = 48)	Total no. of cases according to age distribution (p16 positive case)		
			<40 years	40-60 years	>60 years
Larynx	Papilloma	5	3 (1)	1	1
	Carcinoma	13	1	8 (4)	4
Hypopharynx	Carcinoma	2	0	1	1
Oropharynx	Papilloma	2	2 (1)	0	0
	Carcinoma	2	0	2 (2)	0
Tonsils	Tonsillitis	24	24 (7)	0	0

Eight cases (33.3%) in the tumor group showed positive stain for p16 in IHC. One of two cases with oropharyngeal papilloma and all two cases with oropharyngeal carcinoma stained positive for p16. Both cases with hypopharyngeal ca didn't stain positive for p16. Similarly, of 18 cases with laryngeal tumors, four cases with laryngeal ca and one case with laryngeal papilloma stained positive for p16. All cases were below 60 years. In non tumor group, seven cases (29.1%) showed positive IHC staining for p16 (Table 1).

## DISCUSSION

This study is the first of its kind in Nepal. No study till date has been done in Nepal to detect the prevalence of p16 expression in head & neck tumors. P16 gene is encoded by CDKN2A located in chromosome 9p21 and this is the frequently affected site in the molecular progression of HNSCC. P16 acts by inhibiting CDKN2A which in turn prevents the phosphorylation of Rb protein. Transcription factor E2F1 requires Rb protein to be phosphorylated for it to be dissociated from Rb and thus promote transcription of target genes necessary for G1/S transition.^4^ Thus, p16 is known to slow the cell cycle progression from G1 to S phase. P16 overexpression is considered now a good prognostic factor, specially in cases with OPSCC. It is used as a surrogate marker for HPV infection as it is expressed in most of the cases with positive detection of HPV-16. E6 and E7 oncogenes produced by HPV-16 inactivate tumor suppressor genes p53 and pRb which in turn causes overproduction of p16.^5^ Apart from HPV +ve/p16 +ve tumors, a distinct subgroup of HPV-ve/p16 + ve tumors also exists suggesting a different pathology other than HPV infection resulting in carcinogenesis. While there are plenty of studies on the prognostic role of p16 overexpression in OPSCC, its role in non OPSCC is however understudied. The rationale of our study was thus to estimate the hospital based prevalence of p16 expression in laryngeal and pharyngeal tumors and to compare it with the p16 expression in non tumor site, for which we took tonsillar specimens after tonsillectomy in cases with recurrent acute tonsillitis.

In our study, the majority of cases had laryngeal tumors with 13 having carcinoma of the different subsites and five having papilloma of glottis. P16 staining was positive in four cases with laryngeal ca and all of the cases were male in their 4^th^-6^th^ decades of their life. Only one case with papilloma stained positive for p16. The case was a female in her third decade of her life. All cases in ca group had a history of consumption of cigarettes or tobacco. A study by Orita et al. showed laryngeal papilloma comprised of 44% (34) of total 77 cases of head and neck squamous papilloma. Amongst those cases with laryngeal papilloma, IHC showed positive staining in 64.7% (22) of cases for p16.^6^ Similarly, Laco, et al. in their study for HPV infection and p16 expression in different laryngeal lesions, p16 expression was seen in 18 (78%) of 23 cases with laryngeal papilloma.^7^ In a study from Japan by Kiyuna, et al., only five (5.7%) of the 88 cases with laryngeal ca were p16 positive.^8^ All of those cases were male and also positive for high risk HPV. The cumulative five year survival in this group was relatively higher than p16-ve group. Laco, et al. had relatively higher rate (58%) of positive detection of p16 staining among 24 cases with laryngeal ca. All these cases were also positive for high risk-HPV.^7^ Another study by Hernandez, et al. showed 7.9% cases positive for p16 amongst a total of 101 laryngeal SCC. Although in their study, 32 other cases also showed a diffuse nuclear and cytoplasmic staining, they didn't consider it positive as the staining was seen in <70% of the tumor cells.^9^ Similarly, in a study from China by Galera, et al. 18% of cases with laryngeal squamous cell carcinoma showed immunoreactivity for p16. However, the survival outcomes were similar between p16 positive and negative group.^10^ Young, et al. had 6.5% of cases positive for p16 of 307 cases with laryngeal ca. In contrast to our study, cases with p16+ve tumors were female in majority.^11^

Incidence of hypopharyngeal ca is relatively less compared to the carcinomas of other sites in head and neck region. It accounts upto three to five percent of all HNSCC.^12^ Less has been studied on p16 expression and its role in outcomes of hypopharyngeal ca. There were only two cases of hypopharyngeal ca in our study, both were male and above 40 years. Both of them were p16-ve on IHC and both had history of smoking. Wendt, et al., in their study including 142 cases of hypophayrngeal ca, 109 cases were evaluated for HPV and p16 overexpression. Seven cases were positive for HPV. Of those, four cases were positive for HPV-16 as well as p-16. While there was a statistical co-relation between expression of HPV and overall survival, p16 expression couldn't yield similar statistical significance.^12^ Similarly, Wilson, et al. showed p-16 overexpression in nine (33%) of 27 cases with hypopharyngeal ca. Only one case was positive for HPV. When compared to p16-ve group, there was no statistical difference in mean overall survival, locoregional control and disease free survival.^13^ Similar outcomes were seen in the study by Lee, et al., where although p16 expression was associated with hypopharyngeal ca, it didn't offer any prognostic significance.^14^

Incidence of OPSCC is increasing over past few decades. This increasing incidence is thought to be linked with HPV and is commonly seen in white men in fourth to fifth decades of life.^15^ HPV related oncogenesis is also associated with p16 overexpression due to inhibitory action of E6/E7 oncogenes on p53 and pRb.^2,5^ Association of p16 overexpression in HPV associated OPSCC is well studied and p16 overexpression is often taken as a surrogate maker for HPV infection.^2,16^ In this study, there were only two cases of oropharyngeal carcinoma and both were male and below 60 years. Both were smokers with one having history of tobacco consumption as well. Also, both of the cases stained positive for p16. There was one case with oropharyngeal papilloma who stained positive for p16. The case was a young female with anterior tonsillar papilloma. In a retrospective study by Stephen, et al. p16 was statistically more prevalent in opropharyngeal sites compared to non-oropharyngeal sites in head and neck region. Also, there was improved survival in p16 positive patients for all head and neck subsites.^3^ In a study from China on a large sample of 1470 specimens of OPSCC, p16 overexpression was seen only in 5.51%. Although, the prevalence of p16 in OPSCC was comparatively low, there was a good concordance between HPV detection and p16 overexpression. Majoirty of p16 positive cases were smoker and consumed alcohol.^17^ Liu, et al. in their study demonstrated positive p16 stain in 75 out of 184 cases with OPSCC. HPV 16 and p16 results showed 89% concordance and high risk(HR) HPV and p16 results showed 92% concordance. Cases with HR HPV + ve/p16 + ve, HR HPV-ve/p16 + ve and HR HPV + ve/p16-ve had significantly improved median overall survival compared to HR HPV-ve/p16-ve group.^18^ Similar were the prognostic outcomes in studies by Fischer, et al. and Sedghizadeh, et al.^16^,^19^

While p16 overexpression in malignant tissue is well documented, it is still understudied in non-tumor tissue. In this study, we took tonsillar tissue as non-tumor tissue from cases who underwent tonsillectomy for recurrent acute tonsillitis to see expression of p16. Out of 24 cases, seven (29.1%) cases showed positive stain for p16 in IHC. All cases were below 40 years and four of them were female and three were male. This was a very unique finding. Very few studies have been done and not much have been mentioned in the literatures regarding p16 overexpression in pathologies other than malignancy. A study by Klingenberg, et al. had findings in accordance to ours. A quarter of non-tumor tonsil samples showed p16 overexpression in their study. In only one of those positive cases, HPV DNA was detected.^20^ This suggests a different pathology other than HPV infection in overexpression of p16. The possible explanations for this finding could be infection by other viral or bacterial infection that can upregulate p16 expression or accumulation of p16 in ageing cells.^20^

Small sample size was one of the limitations of our study. Also, we considered positive stain for p16 if there was change in colour of the substrate (DAB) but we didn't quantify the number of cells which stained positive for p16. Another limitation of our study was to not investigate further for HPV DNA in tissues which stained positive for p16. However, as p16 overexpression is considered itself as a good prognostic factor, detection of HPV DNA doesn't offer any additional advantage.

## CONCLUSIONS

Based on the results of our study we could conclude: Both benign and malignant tumors of various subsites of the head and neck region can express p16 and tonsillar tissue affected by recurrent inflammation can also express p16.

A prospective study with large sample from different head and neck site tumors to not only assess p16 expression, but also to assess its implications in the disease course specially for non OPSCC should be done. Also, p16 expression seen in non tumor tissue i.e. tonsil in our study was a very unique finding. This finding also warrants further studies focusing on various factors that can upregulate p16 in normal tissue.

## Conflict of Interest

**None.**
